# Judicial Advice to Medical Practitioners

**Published:** 1844-05

**Authors:** 


*Judicial Advice to Medical Practitioners.*—"In the course of a case which was tried at the Old Bailey yesterday, a medical witness, in giving his evidence, used the word 'tumefaction,' upon which Mr. Justice Coleridge said, 'I suppose by tumefaction you mean swelling.' Witness—'Yes, my lord.' Mr. Justice Coleridge—'Then it would be much better to use plain English, than to speak that sort of mongrel Latin.'" Such is the purport of a paragraph in the *Times* of Wednesday, or rather such is the paragraph itself. Now, we must say, that, if correctly reported, Mr. Justice Coleridge was most absurdly hypercritical; we deny that "tumefaction" is mongrel Latin, or even a pedantic expression, and we think it rather too good that the lawyer should think of correcting the doctor for a fault which the world at large regard as *par excellence* the foible of the gentlemen of the long robe.—*London Medical Gazette.*

